# Toxic Relationships: Characterization of a Putative Virally Encoded Toxin in the Thermophilic Archaeal Fusellovirus SSV1

**DOI:** 10.3390/v18070802

**Published:** 2026-07-21

**Authors:** Jonathan C. Abshier, Patrizia L. Alpapara, Guasåli Tomokane, Kenneth M. Stedman

**Affiliations:** Center for Life in Extreme Environments and Biology Department, Portland State University, Portland, OR 97201, USA; jabshier@pdx.edu (J.C.A.); alpapara@pdx.edu (P.L.A.); guasali@pdx.edu (G.T.)

**Keywords:** viral toxin, archaea, archaeal viruses, extremophiles, toxin–antitoxin systems

## Abstract

Mechanisms for the maintenance of chronic viruses are poorly understood, particularly for archaeal viruses. Here, we identify the product of Sulfolobus spindle-shaped virus 1 (SSV1) ORF a291 as a putative virally encoded toxin required for growth inhibition but dispensable for viral replication and virion production. Viruses lacking ORF a291 replicated their genomes and formed morphologically normal spindle-shaped particles, yet failed to inhibit the growth of uninfected *Saccharolobus solfataricus*. Substitution of residues at a predicted N-terminal signal peptide cleavage site abolished growth suppression without affecting replication, suggesting that secretion is essential for toxin function. Despite primary sequence divergence among fusellovirus toxin candidates, analysis of protein structure predictions revealed a conserved hydrolase-like fold across SSV1, SSV9 and SSV10 toxins. These findings demonstrate functional separation of viral replication and host growth suppression and support a model in which chronic archaeal viruses modulate host competition through antagonistic factors. This work expands the known diversity of putative viral toxins and suggests that fuselloviruses employ conserved strategies to promote persistence in extreme environments.

## 1. Introduction

Viruses are extremely important drivers of microbial evolution and can shape ecological and biogeochemical processes through influencing host fitness, population dynamics, and community structures [1,2,3,4]. In many systems, viruses exert these effects primarily through host cell lysis, thereby reducing host density and releasing cellular contents into the environment to be recycled [3,4]. This lytic paradigm has strongly influenced how viral activity in microbial populations is generally conceptualized [5]. However, ecological models describing virus–host interactions, particularly for lysogenic and chronic viruses, now extend beyond lytic Kill-the-Winner dynamics, where the most abundant hosts are killed, to include Piggyback-the-Winner models, in which viruses preferentially persist in abundant hosts; Kill-the-Competitor strategies, where viral activity suppresses competing host populations; and arms-race models where host defense mechanisms compete with viral anti-defense mechanisms. These are only a few of the diverse strategies by which viruses structure microbial populations [6,7,8].

In contrast to lytic viruses, many archaeal viruses establish chronic infections that do not result in immediate host cell death [7,8,9]. These viruses often exit their hosts through budding or extrusion, allowing infected cells to survive and continue dividing while maintaining viral genomes as integrated and/or episomal DNA [9,10]. These infection strategies raise the question: Can chronic viruses influence host population structure and competition if they do not reduce host density through lysis? In some ecological frameworks, temperate or chronic viral strategies may function analogously to Piggyback-the-Winner or Kill-the-Competitor dynamics, in which viral activity reshapes host competition without necessarily causing widespread lysis [6]. Geothermal environments where archaeal populations and their chronic viruses often dominate microbial communities [8,9,10,11] are excellent systems to study these interactions. Recently, ecosystem structuring via virus-mediated cellular antagonism has been proposed for *Sulfolobus islandicus* and fusellovirus SSV9 [12].

Sulfolobus spindle-shaped virus-1 (SSV1) is an archaeal extremophilic virus [13,14,15] that infects *Saccharolobus* (previously *Sulfolobus*) *solfataricus*, a hyper-thermophilic acidophile that thrives in geothermal hot springs worldwide [16]. Optimal growth conditions for *Saccharolobus* range from 65 °C to 80 °C and a pH of 3.5–4 [16,17]. SSV1, like all fuselloviruses, has a spindle-shaped virion, a morphology characteristic of many archaeal viruses [9,10], and buds from the host cell without killing it [10]. SSV1 has a double-stranded DNA circular genome 15.6 kbp in size [18], and, unlike almost all extremophilic viruses, is genetically tractable [14,19]. SSV1 is very resistant to mutation, with approximately half of the genes in the genome that can be deleted or tolerate the insertion of a 2000 bp transposon that presumably disrupts the function of the gene [19]. Upon infection with SSV1, a copy of the virus genome integrates into the host genome [15], and about five copies of the viral genome are present per cell as episomal DNA [20]. Fusellovirus infection is widespread in *Sulfolobus/Saccharolobus*. Recently, the related fusellovirus SSV9 was shown to provide a selective advantage to infected *Saccharolobus* relative to CRISPR-immune strains presumably via expression of the SSV9 ORF b310 virus-encoded toxin [12,21].

**Figure 1:**
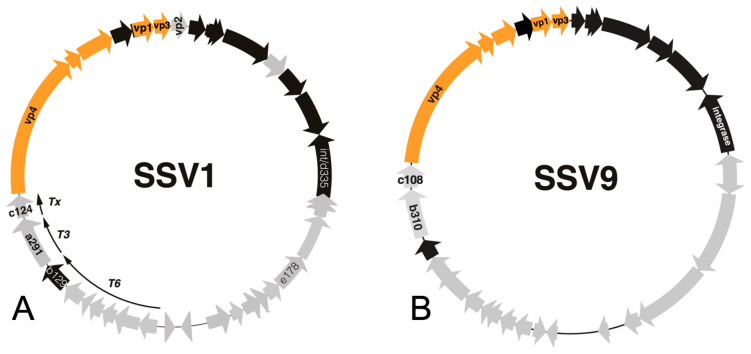
Genome context of putative toxin genes in SSV1 and SSV9. (**A**) SSV1 genome map with labeled open reading frames (ORFs) discussed herein. Universally conserved fusellovirus ORFs are indicated as thick black arrows, while non-conserved ORFs are shown in gray [19]. ORFs encoding structural proteins are highlighted in orange. Thin black arrows indicate transcripts in this region of the genome. (**B**) SSV9 genome map with the same color coding as A [21].

Here, we investigate the product of SSV1 ORF a291, encoded upstream of the SSV1 capsid tail protein VP4 (Figure 1) in the genetically tractable SSV1 model. We tested whether SSV1 viruses lacking a functional ORF a291 could replicate their genomes, form virus particles, or inhibit host growth, as measured by the formation of zones of clearing (halo assays) on lawns of uninfected cells. Taking together our experimental observations and additional sequence and structural analysis of ORF a291, we hypothesize that SSV1 ORF a291 encodes a secreted hydrolase-like toxin that enables infected cells to suppress the growth of uninfected neighbors, promoting SSV1 persistence in *Saccharolobus* populations in extreme environments.

## 2. Materials and Methods

### 2.1. Strains, Media, and Culture Conditions

All experiments were performed using *Saccharolobus solfataricus* strain S441 [22], a strain from Lassen Volcanic National Park in the USA that is an excellent host for SSV1. Cultures were maintained under aerobic conditions at 80 °C in 1× Yeast Sucrose (YS) media (pH 3.5) [15,19]. Liquid cultures were incubated in air with shaking at 150 rpm unless otherwise indicated. Semi-solid plates were made by mixing YS at 2× concentration with molten 1.4% (*w*/*v* in water) Gelrite^®^ (Sigma-Aldrich, St. Louis, MO, USA) and pouring immediately. Plates were allowed to solidify at room temperature overnight prior to use. For the transformation of *S. solfataricus* S441, cells were prepared and electroporated as previously described [15,19]. Cell-free supernatants were prepared from liquid cultures at an OD600 of 0.8 and were centrifuged at 7000× *g* for 10 min at ambient temperature to pellet cellular debris. Supernatants were subsequently filtered through sterile 0.2 µm polyethersulfone (PES) syringe filters (MilliporeSigma, St. Louis, MO, USA) to remove remaining cells and debris. Filtered supernatants were either used immediately for halo assays or stored at 4 °C for future use. For viral passaging experiments, 0.5 to 1 mL of mid-log-phase culture (uninfected or infected) *S. solfataricus* S441 was diluted 1:10 in fresh 1× YS media and incubated at 80 °C with shaking. After 48 h, the same dilution was performed. Three sequential passages were performed to assess viral maintenance.

### 2.2. Virus Constructs Used and Mutagenesis

Previously described SSV1 insertion mutants [19] were grown from laboratory stocks. Targeted deletions of SSV1 ORFs a291 and c124 were constructed in the stand-in wild-type EAI283 shuttle vector (SWT), which contains a stable Tn5 insertion including Kanamycin resistance [19]. Deletions were generated by inverse PCR amplification (LIPCR [19]) of the entire plasmid backbone (10–50 ng of template), excluding the desired open reading frame. Primers were designed flanking SSV1 ORFs a291 or c124 to allow for blunt-end ligation (Table S1). PCR amplification was performed using RepliQa HiFi DNA polymerase (Quantabio, Beverly, MA, USA) under manufacturer-recommended conditions. An amount of 1 µL of amplified products was treated with a Kinase-Ligase-DpnI mixture (KLD; unpublished data) for 1 h at 37 °C with 1U DpnI (New England Biolabs, Ipswich, MA, USA), 1U T4 DNA kinase, 1U DNA ligase in the accompanying DNA ligase buffer (10×) (New England Biolabs, Ipswich, MA, USA). Ligated products were transformed into chemically competent *E. coli* Pir+ cells (Epicentre Biotechnologies, Madison, WI, USA) using a standard heat-shock transformation protocol. Briefly, 2–5 µL of KLD reaction was added to 50 µL competent cells and incubated on ice for 30 min, followed by heat shock at 42 °C for 30 s. Cells were recovered in SOC medium for 1 h at 37 °C with shaking prior to plating on selective LB agar. Plasmids were purified using a GeneJET Plasmid Miniprep Kit (Thermo Scientific, Waltham, MA, USA) and verified by Sanger sequencing (Eurofins Genomics, Louisville, KY, USA) prior to transformation into *S. solfataricus*. Site-directed single nucleotide substitutions at the predicted signal peptide cleavage site of SSV1 ORF a291 (A25L/L26A) were introduced by LIPCR [19]. Primers used are indicated in Supplemental Material (Table S1).

### 2.3. Halo and Plaque Assays

**Figure 2:**
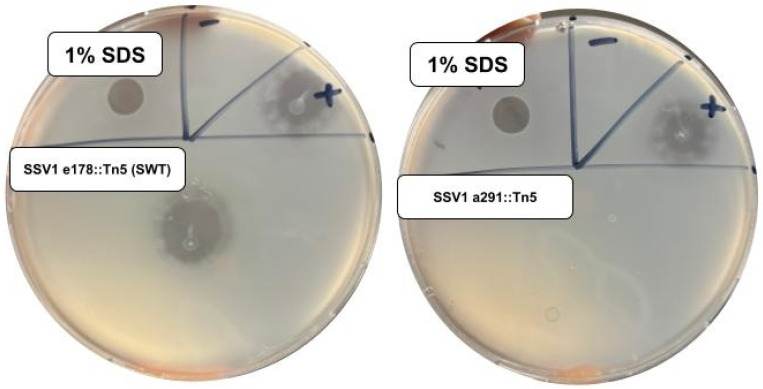
Representative halo assays for growth inhibition of *S. solfataricus* by SSV1. Three microliters of cell-free supernatants from *S. solfataricus* cultures transformed with different SSV1 genomes were spotted onto lawns of uninfected *S. solfataricus* strain S441 and incubated at 80 °C for 72 h. A “+” indicates the quadrant in which supernatant from a known SWT-infected culture was spotted. A “−“ indicates where the supernatant from an uninfected culture was spotted. The label “1% SDS” indicates where 3 µL of 1% Sodium Dodecyl Sulfate was spotted. Bottom half, left panel: 3 µL of culture supernatant from *S. solfataricus* S441 transformed with SWT SSV1 (ORF e178::Tn5) [19]. Bottom half, right panel: 3 µL of culture supernatant from S441 *S. solfataricus* transformed with SSV1 a291::Tn5 (for other halo assays see Supplemental Figure S1).

To assess growth inhibition phenotypes (halo formation), 3 µL aliquots of cell-free supernatants from transformed *S. solfataricus* S441 cultures were spotted onto lawns of uninfected *S. solfataricus* S441 embedded in semi-solid YS medium. Lawns were prepared by mixing mid-log-phase (OD600 = 0.2–0.4, ca. 1.5–2.5 × 10^8^ cells/mL). *S. solfataricus* S441 cultures with YS medium with 0.7% (*w*/*v*) molten Gelrite prior to spreading on pre-warmed YS plates. Plates were allowed to solidify and equilibrate at 80 °C for 30 min before spotting. Plates were incubated for 4–5 days at 80 °C. Visible zones of clearing (halos) were recorded (see Figure 2 and Figure S1). At least 3 biological replicates and 2 technical replicates were performed for each construct. Negative controls included supernatants from uninfected *S. solfataricus* S441 cultures. Positive controls included supernatants from SWT SSV1-transformed cultures. For plaque assays (Figure S2), cell-free supernatants were diluted with YS medium and 1 mL was spread on a lawn of uninfected *S. solfataricus* S441 as above.

### 2.4. PCR Cell-Free Supernatant Analysis

To assess viral genome presence, PCR amplification of the SSV1 structural gene *vp1* and the ORF a291 region was used (Table S1) (see above). Amplification was performed in 25 µL reactions containing 1× Taq buffer, 0.2 mM dNTPs, 0.5 µM forward and reverse primers, 1 U Taq DNA polymerase (New England Biolabs), and 1–3 µL filtered cell-free supernatant as template. Cycling conditions consisted of an initial denaturation at 95 °C for 30 s, followed by 35 cycles of denaturation at 95 °C for 30 s, primer annealing at 56 °C for 60 s, and extension at 68 °C for one minute/kbp of target sequence. A final extension step was performed at 68 °C for 5 min prior to a 10 °C hold, following manufacturer recommendations for Taq DNA polymerase (New England Biolabs).

PCR products were separated on 1% agarose gels stained with ethidium bromide, at a final concentration of 1.0 µg/mL and visualized under UV illumination.

### 2.5. Transmission Electron Microscopy (TEM)

Virions were isolated from cell-free supernatant (see above) by centrifugation at 100,000× *g* for 2 h at 4 °C. Pelleted virions were resuspended in sterile deionized water and adsorbed onto carbon-and formvar-coated 400 mesh copper grids (Ted Pella, Redding, CA, USA) for 2 min. Samples were negatively stained with 2% (*w*/*v*) uranyl acetate for 1 min and examined using a Talos^®^ or Tecnai^®^ transmission electron microscope (FEI, Hillsboro, OR, USA). Spindle-shaped virions were identified based on morphology consistent with previously described SSV1 particles [10,14,15].

### 2.6. Sequence Alignments and Analysis

Since SSV1 ORF a291 had no clear homologs in the NCBI database, 38 published fusellovirus genomes (Supplemental Table S2) were manually inspected, and ORF sequences of around 300 amino acids located upstream of the *vp4* tail protein gene were identified as putative toxin-encoding sequences. Redundant sequences were removed using Geneious Prime (v. 2025.2; Biomatters, Ltd., Auckland, New Zealand) sequence filtering, excluding sequences with >97% amino acid identity. Multiple sequence alignments were generated with ClustalW [23] with default parameters. Signal peptides were predicted using SignalP6.0 [24] (Supplemental Table S3).

### 2.7. Structural Modeling

Predicted protein structures were generated using AlphaFold 3 [25]. Structural homology searches were performed using Foldseek 10 [26], comparing predicted SSV1 ORF a291 models against the Protein Data Bank (PDB) [27]. E-values, TM-scores, and RMSD values were used to quantify structural similarity between predicted toxin models and experimentally determined structures. Structural superimpositions were visualized using ChimeraX 1.10 [28]. All software tools were used with default parameters.

## 3. Results

### 3.1. SSV1 ORF a291 Is a Putative Toxin-Encoding Gene

SSV1 ORF a291 is located in the SSV1 genome between universally conserved ORF b129 and the *vp4* tail protein gene [18,19], in the same genomic context as the SSV9 toxin gene ORF b310 [21]; Figure 1 and toxin loci in SSV11 and SSV13 [12]. However, the sequence of SSV1 ORF a291 is not well-conserved among SSVs, including SSV9 [19]; Figure 1 and see below. Despite this lack of conservation, a 2000 bp transposon insertion in SSV1 ORF a291 generated apparently non-functional SSV1 mutants [19]. Loss of virus function was inferred from the inability of cell-free supernatants from cells transformed with mutant SSV1 genomes to inhibit growth on lawns of uninfected cells [19]. However, growth inhibition could be caused by a virus-encoded toxin and be independent of virus genome replication, virion formation and infectivity. Thus, lack of growth inhibition is not necessarily indicative of lack of virus function. Given the genomic context of SSV1 ORF a291, particularly as compared to SSV9 ORF b310, lack of conservation, and phenotypes of insertion mutants, we decided to investigate SSV1 ORF a291’s potential role as a toxin. Moreover, based on genomic context, SSV1 ORF a291 was predicted by others to encode a toxin [21].

### 3.2. An Intact SSV1 ORF a291 Is Necessary for Host Growth Inhibition

To test the role of SSV1 ORF a291 in infection, we analyzed SSV1 genomes carrying transposon insertions within ORF a291 [19], deletion mutants of ORF a291, and deletions of both ORFs a291 and c124 (the ORF directly downstream of ORF a291 and potential antitoxin, see Discussion). We confirmed previous results [19] that cell-free supernatants from *Saccharolobus solfataricus* strain S441 [22] cultures transformed with virus genomes with transposon insertions within ORF a291 failed to produce a characteristic halo of growth inhibition when spotted on lawns of *S. solfataricus* strain S441 (Figure 2, Table 1). Cell-free supernatants of cultures transformed with SSV1 genomes with an intact ORF a291, but containing the same transposon insertion in ORF e178 [19], used as a replicative vector and control (hereafter referred to as Stand-in wild-type, SWT), did cause halos (Figure 2, Table 1). We also made in-frame deletions of SSV1 ORF a291 and SSV1 ORFs a291 and c124 in the SWT background to ensure that the altered phenotypes were not due to the transposon insertion in these ORFs. Both deletion mutants also failed to inhibit the growth of uninfected cells (Figure 2, Table 1, Supplemental Figure S1).

**Table 1:** Genotype and phenotype summary of SSV1 ORF a291 mutants.

Genotype/Mutation Description	Halo Formation ^†^	Replication (PCR) ^‡^	Virions Observed ^§^
SSV1 e178::Tn5 * (SWT)	+	+	+
SSV1 a291::Tn5 *	−	+	+
SSV1 e178::Tn5 Δa291	−	+	+
SSV1 e178::Tn5 Δa291–c124	−	+	+
SSV1 e178::Tn5 a291 (A25L/L26A)	−	+	+

**Figure 3:**
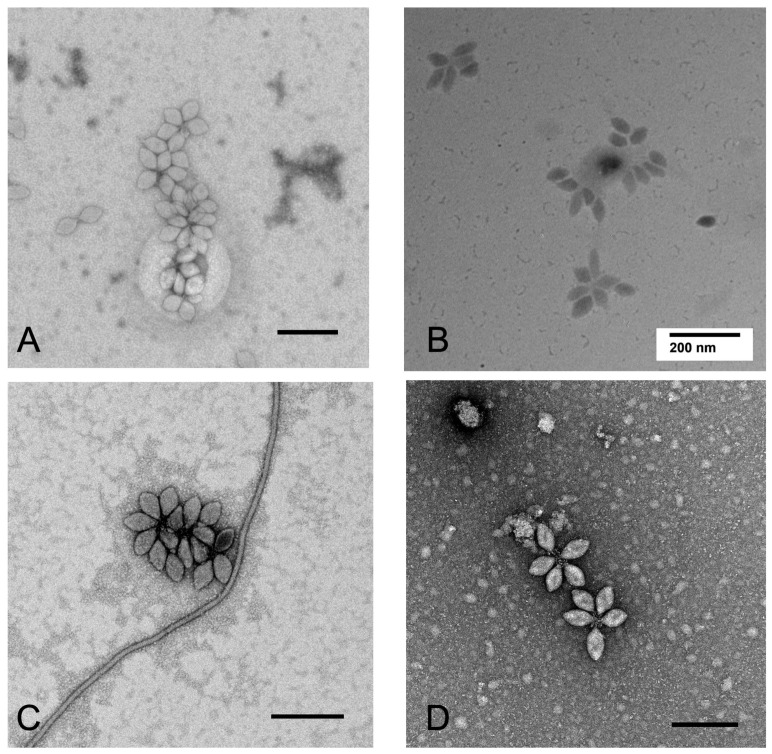
Transmission electron micrographs of virions from *S. solfataricus* S441 cultures transformed with SSV1 viral genomes. Virions were stained with 1% uranyl acetate and imaged with a FEI Tecnai transmission electron microscope or a FEI Talos transmission electron microscope. Scale bars represent 200 nm. (**A**) Virions from SWT SSV1, (**B**) virions from SSV1 a291 transposon insertion mutant (EAI254 SSV1 a291::Tn5), (**C**,**D**) virions from single and double deletions in SWT (ORFs a291 and a291-c124, respectively).

* For construction of mutants, see [19]. ^†^ Negative (−) halo formation indicates no halo formation from 3 transformations for which positive transformation controls generated halos (see Figure 2). ^‡^ Replication phenotype “+” indicates that SSV1-specific PCR amplicons were generated from cell-free supernatants of transformed cultures. ^§^ Virions observed “+” indicates that typical fusellovirus virions were observed in cell-free supernatants by transmission electron microscopy (Figure 3).

### 3.3. SSV1 Genomes with Disrupted or Deleted ORF a291 Still Replicate Their Genomes

To determine if SSV1 genomes lacking a functional ORF a291 were able to replicate and infect uninfected *S. solfataricus*, genome replication by mutant SSV1 genomes was checked using end-point PCR. PCR with primers targeting the SSV1 major structural gene *vp1* [29] and ORF a291 was performed on cell-free supernatants of *S. solfataricus* cultures transformed with different SSV1 genomes. SSV1-specific amplicons were generated from cell-free supernatants of *S. solfataricus* transformed with SWT SSV1, SSV1 genomes with transposon insertions in ORF a291, as well as genomes with deletions of ORF a291 and both ORFs a291 and c124 (Table 1). As controls, PCR from cell-free supernatants from cells mixed with DNA, but not electroporated, as well as cells without added DNA, did not generate SSV1-specific amplicons, thereby excluding residual DNA contamination. Cultures from cells transformed with a transposon insertion at bp 8633 in SSV1 ORF a291 were passaged 3 times (see methods) without losing the ability to amplify SSV1-specific genes from cell-free supernatants, indicating that viruses generated by these mutants were stably maintained.

### 3.4. SSV1 Genomes with Disrupted or Deleted ORF a291 Can Form Virions

To determine if SSV1 ORF a291 is important for virion production, cell-free supernatants from cells transformed with SWT SSV1, and SSV1 virus genomes with transposon insertions in ORF a291, as well as SSV1 genomes with deletions of ORF a291 and both ORFs a291 and c124, were screened for typical spindle-shaped virions using transmission electron microscopy (TEM). Cell-free supernatants from *S. solfataricus* transformed with SWT SSV1, SSV1 with a Tn5 insertion in ORF a291, and deletions of SSV1 ORF a291 and deletion of SSV1 ORFs a291 and c124 all contained typical fusellovirus-like particles (Table 1, Figure 3). This indicates that SSV1 ORF a291 is not necessary to generate virus particles.

### 3.5. Putative Toxin Gene Sequences in Fuselloviruses Are Not Well Conserved

**Figure 4:**
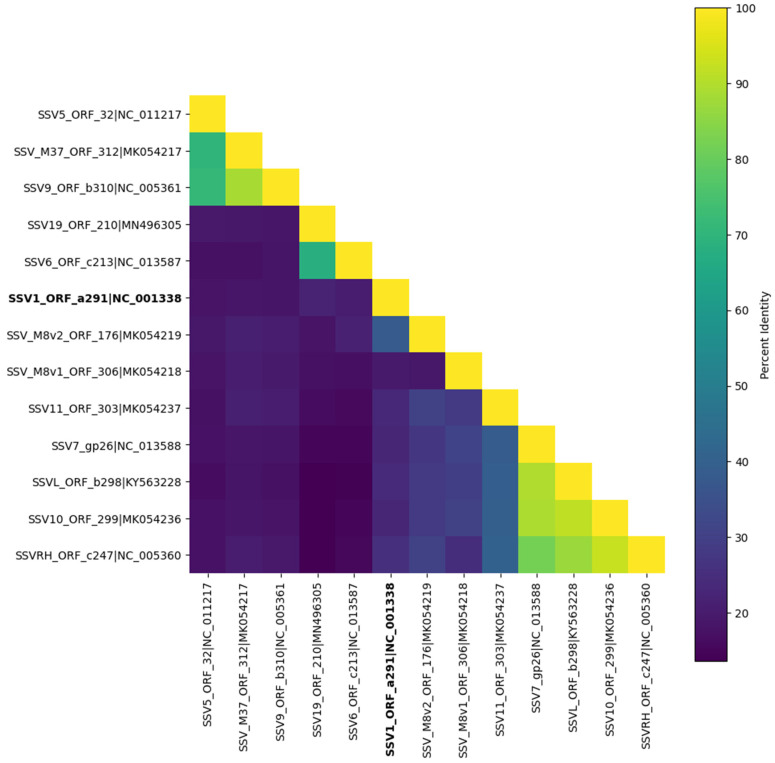
Percent identity matrix of all known non-redundant fusellovirus candidate toxin homologs. Pairwise amino-acid percent identities between fusellovirus toxin homologs were calculated using ClustalW and visualized using RStudio v 4.5.2 [30] and pheatmap package v 1.0 [31]. Each cell shows the percent identity for the corresponding toxin pair, with warmer colors indicating higher sequence identities. Diagonal values represent self-comparisons. SSV1_ORF_a291 is highlighted in bold. Accession numbers for all sequences are listed next to fusellovirus ORF names.

In previous analyses of SSV1 ORF a291, no clear homologs were found in other SSV genomes using BLASTp Version 2.5.0+ (e < 0.001) [19]. However, there is syntenic conservation of the putative toxin gene locus among published SSV genomes, and many SSV genomes have ORFs of approximately 300 amino acids at that locus (Figure 4) [21]. Therefore, we collected all 13 non-redundant putative toxin-encoding sequences from the 38 available SSV genomes and performed pairwise comparisons between them (see Section 2 methods). Only one other putative fusellovirus toxin sequence was more than 30% identical in amino acid sequence to SSV1 ORF a291, indicating that these proteins are highly divergent despite their conserved genomic context. Notably, SSV9 ORF b310, which encodes the first discovered fusellovirus-encoded toxin, was only 18.7% identical in amino acid sequence to SSV1 ORF a291 (Figure 4).

### 3.6. Alignment of Amino Acid Sequences of Putative Fusellovirus Toxins Indicates the Presence of a Conserved Signal Peptide and Multiple Related Toxins

**Figure 5:**
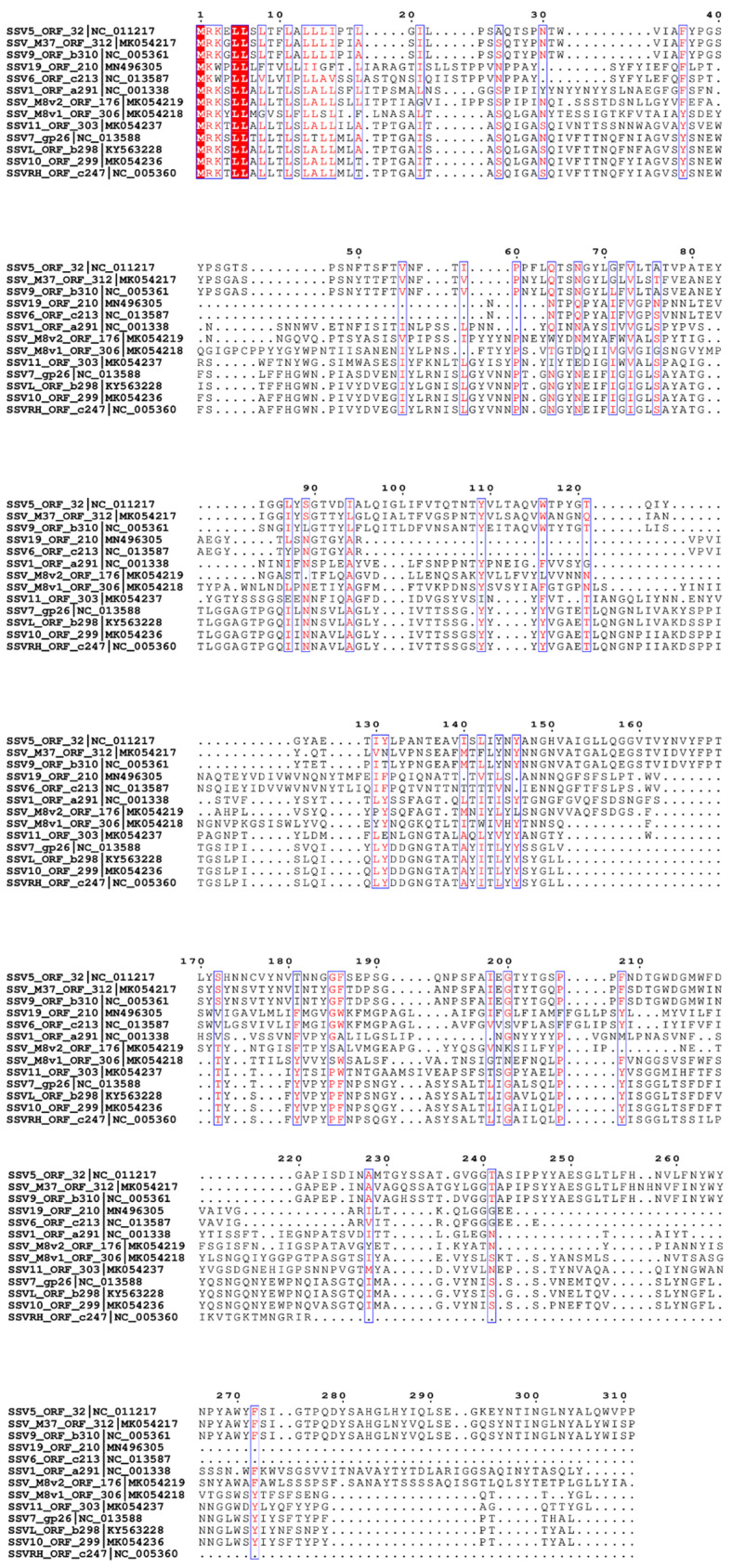
Multiple sequence alignment of all known non-redundant fusellovirus toxin candidates. A multiple sequence alignment of putative fusellovirus toxin proteins was aligned with CLUSTALW and visualized using ESPript 3.0. Residues conserved in all sequences have a red background, with similar residues indicated by red text and blue boxed highlighting. Sequence identifiers correspond to the viral toxin Genbank protein ID and associated open reading frame, e.g., SSV1_ORF_a291|NC_001338.

In order to determine if there were conserved sequence motifs in the non-redundant putative SSV toxins, their amino acid sequences were aligned using ClustalW [23] with default parameters, and the resulting alignment was visualized in ESPript3.0 [32] (Figure 5). The only amino acids that are 100% conserved are at the N-terminus. Moreover, sequences in this region were generally much more conserved than in other parts of the sequence (Figure 5). Interestingly, this N-terminal region corresponds to a predicted signal peptide in all of the putative toxin sequences, as determined by SignalP 6.0 [24] (Supplemental Table S3).

### 3.7. Changing the Putative Signal Peptide Cleavage Site of SSV1 ORF a291 Eliminates Toxin Activity

To determine if the predicted signal peptide was important for the function of SSV1 ORF a291, we switched the encoded amino acids at positions 25 and 26 in SWT SSV1 ORF a291, A25L/L26A, using site-directed mutagenesis. This should eliminate cleavage by the *Saccharolobus* signal peptidase [24]. We then tested this mutant construct as above for causing growth inhibition, genome replication and virion formation. We found that this mutant did not cause growth inhibition but still replicated its genome and formed virions in a manner similar to the complete deletion of SSV1 ORF a291 (Figure 2 and Supplemental Figure S1, Table S1), indicating that signal peptide cleavage and secretion are essential for the function of the toxin.

### 3.8. The SSV1 ORF a291 Toxin and Other SSV Putative Toxin Proteins Share a Conserved Hydrolase-like Fold Despite Extreme Sequence Divergence

**Figure 6:**
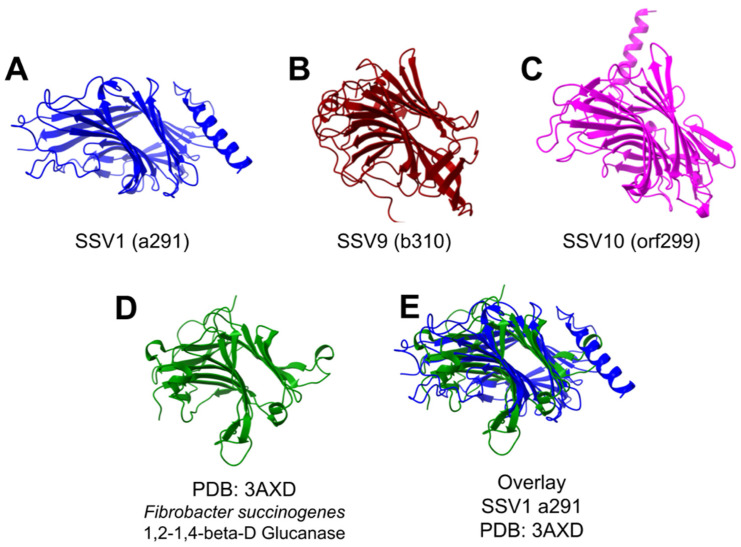
(**A**–**C**) Alphafold3 structural predictions of SSV putative toxin proteins. Models of SSV1 a291 ((**A**) blue), SSV9 b310 ((**B**) maroon), and SSV10 orf299 ((**C**) magenta). (**D**) Crystal structure of *Fibrobacter succinogenes*, 1,3-1,4-beta-D-glucanase (PDB: 3AXD). The 3AXD structure (green) has a canonical hydrolase fold [34]. (**E**) Matchmaker (ChimeraX [28]) of SSV1 a291 (blue) with the hydrolase 3AXD (green), reveals a highly similar overall fold (RMSD 10.04 Å, TM-score = 0.453).

In order to predict the mechanism of action of the putative toxins, Alphafold3 models [25] were constructed for ORFs a291 (SSV1), b310 (SSV9) and 299 (SSV10) (Figure 6). SSV10 is a fusellovirus from Lassen Volcanic National Park, USA, that is also genetically tractable [33]. SSV10 ORF 299 is only 22.9% identical to SSV1 ORF a291 (Figure 4 and Figure 5). All of these models contained a beta-sheet core flanked by short alpha-helices and surface loops (Figure 6) with high confidence. Despite low (>25%) amino acid sequence identity between these sequences, their predicted structures are extremely similar (Figure 6A–C). Foldseek [26] identified highly statistically significant structural matches in the PDB100 database [27] for all three predicted structures. The top match for SSV1 ORF a291, *Fibrobacter succinogenes*, 1,3-1,4-beta-D-glucanase PDB: 3AXD (E-Value 9.86 × 10^−24^), has a very similar fold to the putative SSV toxin structures (Figure 6D). Superimposition of the X-ray crystallography-derived structure 3AXD on the predicted structure of SSV1 ORF a291 (Figure 6E) demonstrates that SSV putative toxins have a canonical hydrolase-like structure.

## 4. Discussion

### 4.1. SSV1 ORF a291 Is Dispensable for Viral Replication but Required for Growth Suppression

Contrary to previous reports [19], PCR amplification of viral genes from cell-free supernatants, even after passaging, indicated that ORF a291-deficient SSV1 viruses replicate (Table 1), and TEM analysis revealed virion morphology very similar to wild-type SSV1 (Table 1, Figure 3). These findings indicate that SSV1 ORF a291 is not required for genome replication and virion assembly. However, these experiments are not quantitative, so viruses lacking ORF a291 could be less fit than the wild-type virus. This possibility will be tested in future experiments. However, it is clear that an intact ORF a291 is required for SSV1 to inhibit the growth of uninfected *S. solfataricus*. (Table 1, Figure 2). This functional separation indicates that SSV1 ORF a291 does not serve a structural or replicative role but instead may control host population dynamics. Accessory metabolic genes (AMGs) encoded in some viruses often mediate ecological interactions without being required for viral reproduction [35,36,37], but generally affect the metabolism of the infected host cell, not other cells [6].

### 4.2. Reinterpreting Halo Formation

Plaque formation on host cells has traditionally been interpreted as a proxy for viral infection and replication, particularly in lytic bacteriophage systems where clear plaques result from cycles of infection and host cell lysis [3,4]. However, many viruses do not form clear plaques. Filamentous bacteriophages such as M13 establish chronic, non-lytic infections yet still produce plaques due to reduced host growth and continuous virion extrusion rather than cell lysis [38]. Temperature bacteriophage form turbid plaques due to bacteriophage resistance by lysogens. Some bacteriophage form “Bulls-eye” plaques with a central area of clearing surrounded by turbidity, followed by an area or halo of clearing [39]. Halo formation by bacteriophage is often thought to be due to the production of enzymes that degrade cellular components [39]. SSV1 was first reported to form turbid plaques with a halo when infecting *Saccharolobus* (previously *Sulfolobus*) *solfataricus* [15].

A rapid method of screening multiple viruses under identical conditions is by spotting a small amount of virus-containing liquid on a lawn of uninfected host cells and observing growth inhibition, also known as “halo” assays or spot testing [39]. This method is often used for fuselloviruses [13], since plaque assays for SSVs are notoriously challenging (see Supplemental Figure S2) [21]. We also use these assays to increase sensitivity since multiple viruses are present in the cell-free supernatant spotted on lawns. We previously interpreted positive halo assays as reflecting direct inhibition of host growth due to virus reproduction [19].

However, disruption or deletion of SSV1 ORF a291 abolished halo formation (Figure 2; Table 1), yet viruses with these mutations appear to replicate their genomes (Table 1) and produce morphologically normal spindle-shaped virions (Figure 3). We infer that halo, and by extension plaque formation, SSV1 reflects virus-encoded toxin-mediated growth suppression rather than merely viral infection. Together, these results are consistent with the presence of a secreted protein factor that is sufficient to induce host growth suppression. Interestingly, SSV9, virus-free supernatants of SSV9-infected cells, and cell-free supernatants from *S. islandicus* cells overexpressing only SSV9 ORF b310 cause complete clearing of *Saccharolobus islandicus* lawns, indicating that this toxin kills uninfected cells [12,21].

### 4.3. SSV Toxin Evolutionary Divergence with Conserved Secretion-Dependent Function

Comparative sequence analysis revealed minimal amino acid identity among putative fusellovirus toxins (Figure 4), including only 18.7% identity between SSV1 ORF a291 and the previously examined SSV9 toxin b310 [12] and 22.92% identity in ORF 299 (putative toxin) of SSV10. Despite this divergence, putative toxin genes are encoded upstream of the tail protein gene *vp4* across fusellovirus genomes, suggesting that functional constraints maintain genomic position [21] (Figure 1). Moreover, the conservation of only putative signal peptides (Figure 5) between these putative toxin genes, despite rapid primary sequence evolution in the remainder of the protein, indicates that secretion is critical for toxin function. Overexpression of a truncated SSV9 ORF b310 supports this interpretation [21]. Substitution of residues at the predicted signal peptide cleavage site abolished halo formation by SSV1 while preserving viral genome replication and virion production (Table 1, Figure 2), similar to the complete deletion of ORF a291. Together, these findings indicate that fusellovirus toxins exhibit extensive sequence divergence while maintaining secretion-dependent function. Other SSV toxins were also predicted to be secreted. Overexpressed SSV9 ORF b310 in the absence of SSV9 is found in cell-free supernatants, indicating secretion. Finally, protein processing of other SSV toxins was predicted but was not directly demonstrated [21].

### 4.4. Structural Conservation of a Hydrolase-like Fold Across Divergent Fusellovirus Toxins

Despite extreme sequence divergence (Figure 4 and Figure 5), AlphaFold 3 predictions indicate that fusellovirus toxins adopt a conserved beta-sheet-rich fold flanked by alpha-helices and surface loops (Figure 6A–C) [25]. As identified with FoldSeek [26], the predicted structures of these toxins are strikingly similar to a bacterial glucanase (Figure 6), an enzyme that hydrolyzes structural polysaccharides. Such enzymes typically cleave glycosidic bonds and modify extracellular substrates. The predicted SSV toxin structures share the canonical beta-sheet-rich fold of this enzyme class, suggesting potential catalytic or substrate-binding activity [25].

The preservation of structural architecture in the absence of detectable sequence similarity reflects functional constraints of the three-dimensional structure rather than primary sequence, a common feature of protein evolution [40,41,42]. Although the precise substrate remains unknown, this hydrolase-like architecture raises the possibility that these toxins act on extracellular targets, potentially altering cell surface components to suppress the growth of neighboring cells.

### 4.5. Toxin–Antitoxin Systems and Viral Population Control

Toxin–antitoxin (TA) systems, also known as addiction modules, are well known in bacteria and archaea [43,44]. TA systems typically consist of a stable toxin and a labile antitoxin encoded as adjacent genes. Loss of the TA locus often leads to toxin-mediated growth arrest or cell death, thereby stabilizing maintenance of the genetic element [43,44]. Although TA systems are well characterized in bacterial chromosomes and plasmids, their roles in archaeal viruses remain largely unexplored [45,46]. Interestingly, SSV1 encodes a non-conserved ORF just downstream of ORF a291, ORF c124, and similar genome orientations of possible antitoxins with putative toxin genes are found in other SSVs [19,21] (Figure 1). However, deletion of this ORF does not change the phenotype of SSV1 ORF a291 deletions (Table 1), nor does an insertion in this ORF in the presence of ORF a291 change halo formation [19]. No difference was seen with overexpression of the corresponding putative antitoxin in SSV9 in the presence of the SSV9 ORF b310 toxin [21]. Thus, the existence and function of a putative antitoxin in SSVs remains to be determined. Interestingly, the presence of the toxin appears to protect cells from the activity of the toxin [21].

### 4.6. Ecological Implications: Growth Suppression as a Persistence Strategy

**Figure 7:**
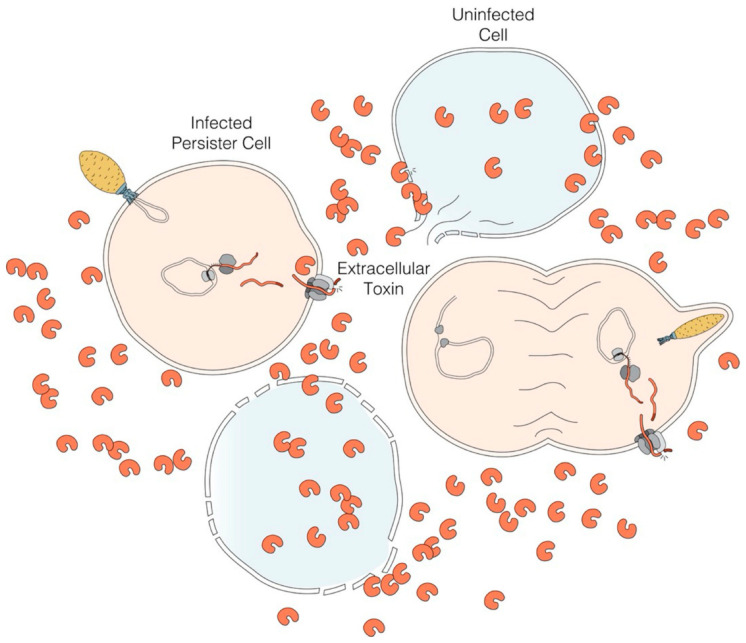
Model of fusellovirus-mediated toxin production dynamics and population-level outcomes. Pink *S. solfataricus* cells are infected with yellow SSVs, which replicate their genomes and produce yellow virions. Putative toxin genes are transcribed and translated (red line). The putative virus-encoded toxins (red C) are secreted and inhibit the growth of uninfected cells (blue). The mechanism of growth inhibition is unknown. Pink infected cells are resistant to the effects of the toxin and outcompete uninfected cells.

These findings indicate that SSV1 ORF a291 encodes a secreted toxin that suppresses the growth of neighboring uninfected cells while leaving viral replication and virion production intact. We propose a model (Figure 7) in which SSV1 establishes chronic infection in *S. solfataricus* that enables secretion of the SSV1 ORF a291 product into the extracellular environment, suppressing the growth of uninfected cells while infected cells persist. This framework is supported by prior work on SSV9-infected *S. islandicus*, in which SSV9 encodes the extracellular toxin b310 that kills uninfected hosts, particularly when overexpressed [12,21]. These toxin genes are conserved upstream of the *vp4* structural gene across multiple fuselloviruses, suggesting functional conservation despite sequence divergence (Figure 4).

Unlike lytic viruses that reduce host density through destruction and nutrient release [3,4], SSV1 appears to modulate competitive dynamics without eliminating its host in a virus–host mutualism similar to SSV9 [12]. Our data support a mechanism in which growth suppression of uninfected cells enhances the relative fitness of infected hosts.

In resource-limited geothermal environments, where microbial diversity is low and competition is intense [11], even modest suppression of competitors may promote long-term virus–host mutualism [12]. This strategy parallels addiction-module theory, in which maintenance of a genetic element confers a selective advantage through toxin-mediated enforced maintenance of the genetic element [44,47]. However, fusellovirus toxins appear to extend this principle beyond maintenance of the element to control of the growth of uninfected cells in the community. Image credit Rita Clare/Scivetica.

## Data Availability

The original contributions presented in this study are included in the article/Supplementary Material. Further inquiries can be directed to the corresponding author.

## Supplementary Materials

The following supporting information can be downloaded at: https://www.mdpi.com/article/10.3390/v18070802/s1. Table S1: Primers used in this study; Table S2: Amino acid sequences of fusellovirus toxin homologs; Table S3: Signal peptide predictions for fusellovirus toxin homologs; Figure S1: Representative growth inhibition phenotypes of SSV1 mutants; Figure S2: Plaque formation by SSV1 in S441.
