# Supplementary material for: Toxic Relationships: Characterization of a Putative Virally Encoded Toxin in the Thermophilic Archaeal Fusellovirus SSV1
Source: Viruses. 2026 Jul 21;18(7):802. doi: 10.3390/v18070802 (PMC13431529; doi:10.3390/v18070802)
Supplement: Supplementary file 1 [file viruses-18-00802-s001.zip › viruses-4377897-supplementary.pdf]

Supplemental Materials

Supplemental Table S1. Primers used in this study.

Primers were used for screening viral replication and for mutagenesis of SSV1 constructs as described in materials and methods.

Primer	Sequence (5’-3’)	Note
Vp1full_F	GCCGATATTGACCAAGGATG	Forward primer to screen Vp1 gene for replication (SSV1 major capsid protein) in cell-free supernatant.
Vp1full_R	ATATCTTATACGCCACGACT	Reverse primer to screen Vp1 gene for replication (SSV1 major capsid protein) in cell-free supernatant.
A291_qPCR_F	TGCCCGCTTTGTCTAATGCCCT	Forward primer to screen for toxin-antitoxin region (C-terminus ORF b129 through N-terminus of Vp4) for replication in cell-free supernatant.
TK qPCR Vp4 1R	GAGTAGTTCGTACGGTTCCTCC	Reverse primer to screen for toxin-antitoxin region (C-terminus ORF b129 through N-terminus of Vp4) for replication in cell-free supernatant.
Q5SDM_A291Del-F	GCAAAATCTTTTTTTACCTCTTTTTAAATC	Forward primer used in long inverse PCR (LIPCR) to delete ORF a291.
Q5SDM_A291Del_R	TGTAAGCGAAAAAGAAGG	Reverse primer used in LIPCR to delete ORF a291 and to delete ORF c124 from subsequent Δa291 mutant. Also used as reverse primer for A291 25/26 Arg/Lys F primer.
A291_25/26 Arg/Lys (L/A) F	ACCATCGATGagaaaaAATTCTGGCGGTTC	Forward primer to substitute cleavage sequence at position 25/26 in ORF a291 A/L -> L/A
SSV1 c124 Del F	CCCTTTTTAAAGCCATAAATTT	Forward primer to delete ORF c124 from Δa291 mutant.

Supplemental Table S2. Amino acid sequences of fusellovirus toxin homologs.

Sequences used for multiple sequence alignment and comparative analyses.

Name	Sequence
SSV11	MRKTLLALLTLSLALLILATPTGAITASQIGASQIVNTTSSNNWAGVAYSVEWRSWF<br>TNYWGSIMWASESIYFKNLTLGYISYPNYIYTEDIGIWVALSPAQIGYGTYSSSGSEE<br>NNFIQAGFDIDVGSYVSINYFVTTIANGQLIYNNENYVPAGNPTYLDMFLENLGNGT<br>ALAQLYVYYANGTYWTITIYTSIPWTNTGAAMSIVEAPSFSTSGPYAELPYVSGGMI<br>HFTFSYVGSDGNEHIGPSNNPVGTMYADVYVLNEPSTYNVAQAQIYNGWANNNGG<br>WDYLYQFYYPGAGQTTYGL*
SSV10	MRKTLLALLTLSLALLMLTTPTGAITASQLGANQIVFTTNQFYIAGVSYSNEWFSAF<br>FHGWNPIVYDVEGIYLRNISLGYVNNPNGNGYNEIFIGIGLSAYATGTLGGAGTPGQI<br>INNAVLAGLYIVTTSSGSYYYYVGAETLQNGNPIIAKDSPPITGSLPISLQIQLYDDGN<br>GTATAYITLYYSYGLLTYSFYVPYPFNPSQGYASYSALTLIGAILQLPYISGGLTSFDFTY<br>QSNGQNYEWPNQVASGTQIMAGVYNISSSPNEFTQVSLYNGFLNNGLWSYIYSFTY<br>PYPTTYAL*
SSV NL13.C01.04.SSV	MRKTLLALLTLSLALLMLTTPTGAITASQLGANQIVFTTNQFYIAGVSYSNEWFSAF<br>FHGWNPIVYDVEGIYLRNISLGYVNNPNGNGYNEIFIGIGLSAYATGTLGGAGTPGQI<br>INNAVLAGLYIVTTSSGSYYYYVGAETLQNGNPIIAKDSPPITGSLPISVQTYLYDDGN<br>GTATAYITLYYSYGLLTYSFYVPYPFNPSQGYAAYSALTLIGAILQLPYISGGLTSFDFTY<br>QSNGQNYEWPNQVASGTQIMAGVYNISSSPNEFTQVSLYNGFLNNGLWSYIYSFTY<br>PYPTTHAL*
SSV NL03.C02.05.SSV	MRKTLLALLTLSLALLMLTTPTGAITASQLGANQIVFTTNQFYIAGVSYSNEWFSAF<br>FHGWNPIVYDVEGIYLRNISLGYVNNPNGNGYNEIFIGIGLSAYATGTLGGAGTPGQI<br>INNAVLAGLYIVTTSSGSYYYYVGAETLQNGNPIIAKDSPPITGSLPISVQTYLYDDGN<br>GTATAYITLYYSYGLLTYSFYVPYPFNPSQGYASYSALTLIGAILQLPYISGGLTSFDFTY<br>QSNGQNYEWPNQVASGTQIMAGVYNISSSVNEFTQVSLYNGFLNNGLWSYIYSFTY<br>PYPTTHAL*
SSV NL03.C02.01.SSV	MRKTLLALLTLSLALLMLTTPTGAITASQLGANQIVFTTNQFYIAGVSYSNEWFSAF<br>FHGWNPIVYDVEGIYLRNISLGYVNNPNGNGYNEIFIGIGLSAYATGTLGGAGTPGQI<br>INNAVLAGLYIVTTSSGSYYYYVGAETLQNGNPIIAKDSPPITGSLPISVQTYLYDDGN<br>GTATAYITLYYSYGLLTYSFYVPYPFNPSQGYASYSALTLIGAILQLPYISGGLTSFDFTY<br>QSNGQNYEWPNQVASGTQIMAGVYNISSSVNEFTQVSLYNGFLNNGLWSYIYSFTY<br>PYPTTHAL*
SSV NL01B.C01.24.SSV	MRKTLLALLTLSLALLMLTTPTGAITASQLGANQIVFTTNQFYIAGVSYSNEWFSAF<br>FHGWNPIVYDVEGIYLRNISLGYVNNPNGNGYNEIFIGIGLSAYATGTLGGAGTPGQI<br>INNAVLAGLYIVTTSSGSYYYYVGAETLQNGNPIIAKDSPPITGSLPISVQTYLYDDGN<br>GTATAYITLYYSYGLLTYSFYVPYPFNPSQGYASYSALTLIGAILQLPYISGGLTSFDFTY<br>QSNGQNYEWPNQVASGTQIMAGVYNISSSVNEFTQVSLYNGFLNNGLWSYIYNFTY<br>PYPTTHAL*
SSV NL01B.C01.22.SSV	MRKTLLALLTLSLALLMLTTPTGAITASQLGANQIVFTTNQFYIAGVSYSNEWFSAF<br>FHGWNTIVYDVEGIYLRNISLGYVNNPNGNGYNEIFIGIGLSAYATGTLGGAGTPGQI<br>INNAVLAGLYIVTTSSGSYYYYVGAETLQNGNPIIAKDSPPITGSLPISVQTYLYDDGN<br>GTATAYITLYYSYGLLTYSFYVPYPFNPSQGYAAYSALTLIGAILQLPYISGGLTSFDFTY<br>QSNGQNYEWPNQVASGTQIMAGVYNISSSPNEFTQVSLYNGFLNNGLWSYIYSFTY<br>PYPTTHAL*
SSV NL01B.C01.20.SSV	MRKTLLALLTLSLALLMLTTPTGAITASQLGANQIVFTTNQFYIAGVSYSNEWFSAF<br>FHGWNPIVYDVEGIYLRNISLGYVNNPNGNGYNEIFIGIGLSAYATGTLGGAGTPGQI<br>INNAVLAGLYIVTTSSGSYYYYVGAETLQNGNPIIAKDSPPITGSLPISVQTYLYDDGN<br>GTATAYITLYYSYGLLTYSFYVPYPFNPSQGYAAYSALTLIGAILQLPYISGGLTSFDFTY

	QSNGQNYEWPNQVASGTQIMAGVYNISSSPNEFTQVSLYNGFLNNGLWSYIYSFTY PYPTTHAL*
SSV NL01B.C01.18.SSV	MRKTLLALLTLSLALLMLTTPTGAITASQLGANQIVFTTNQFYIAGVSYSNEWFSAF FHGWNPIVYDVEGIYLRNISLGYVNNPNGNGYNEIFIGIGLSAYATGTLGGAGTPGQI INNAVLAGLYIVTTSSGSYYYYVGAETLQNGNPIIAKDSPPITGSLPISVQTYLYDDGN GTATAYITLYYSYGLLTYSFYVPYPFNPSQGYAAYSALTLIGAILQLPYISGGLTSFDFTY QSNGQNYEWPNQVASGTQIMAGVYNISSSPNEFTQVSLYNGFLNNGLWSYIYSFTY PYPTTHAL*
SSV NL01B.C01.14.SSV	MRKTLLALLTLSLALLMLTTPTGAITASQLGANQIVFTTNQFYIAGVSYSNEWFSAF FHGWNTIVYDVEGIYLRNISLGYVNNPNGNGYNEIFIGIGLSAYATGTLGGAGTPGQI INNAVLAGLYIVTTSSGSYYYYVGAETLQNGNPIIAKDSPPITGSLPISVQTYLYDDGN GTATAYITLYYSYGLLTYSFYVPYPFNPSQGYAAYSALTLIGAILQLPYISGGLTSFDFTY QSNGQNYEWPNQVASGTQIMAGVYNISSSVNEFTQVSLYNGFLNNGLWSYIYSFTY PYPTTHAL*
SSV NL01B.C01.13.SSV	MRKTLLALLTLSLALLMLTTPTGAITASQLGANQIVFTTNQFYIAGVSYSNEWFSAF FHGWNPIVYDVEGIYLRNISLGYVNNPNGNGYNEIFIGIGLSAYATGTLGGAGTPGQI INNAVLAGLYIVTTSSGSYYYYVGAETLQNGNPIIAKDSPPITGSLPISVQTYLYDDGN GTATAYITLYYSYGLLTYSFYVPYPFNPSQGYAAYSALTLIGAILQLPYISGGLTSFDFTY QSNGQNYEWPNQVASGTQIMAGVYNISSSPNEFTQVSLYNGFLNNGLWSYIYSFTY PYPTTHAL*
SSV NL01B.C01.07.SSV	MRKTLLALLTLSLALLMLTTPTGAITASQLGANQIVFTTNQFYIAGVSYSNEWFSAF FHGWNPIVYDVEGIYLRNISLGYVNNPNGNGYNEIFIGIGLSAYATGTLGGAGTPGQI INNAVLAGLYIVTTSSGSYYYYVGAETLQNGNPIIAKDSPPITGSLPISVQTYLYDDGN GTATAYITLYYSYGLLTYSFYVPYPFNPSQGYASYSALTLIGAILQLPYISGGLTSFDFTY QSNGQNYEWPNQVASGTQIMAGVYNISSSVNEFTQVSLYNGFLNNGLWSYIYNFTY PYPTTHAL*
SSV NL01B.C01.06.SSV	MRKTLLALLTLSLALLMLTTPTGAITASQLGANQIVFTTNQFYIAGVSYSNEWFSAF FHGWNPIVYDVEGIYLRNISLGYVNNPNGNGYNEIFIGIGLSAYATGTLGGAGTPGQI INNAVLAGLYIVTTSSGSYYYYVGAETLQNGNPIIAKDSPPITGSLPISVQTYLYDDGN GTATAYITLYYSYGLLTYSFYVPYPFNPSQGYASYSALTLIGAILQLPYISGGLTSFDFTY QSNGQNYEWPNQVASGTQIMAGVYNISSSVNEFTQVSLYNGFLNNGLWSYIYNFTY PYPTTHAL*
SSV NL01B.C01.05.SSV	MRKTLLALLTLSLALLMLTTPTGAITASQLGANQIVFTTNQFYIAGVSYSNEWFSAF FHGWNPIVYDVEGIYLRNISLGYVNNPNGNGYNEIFIGIGLSAYATGTLGGAGTPGQI INNAVLAGLYIVTTSSGSYYYYVGAETLQNGNPIIAKDSPPITGSLPISVQTYLYDDGN GTATAYITLYYSYGLLTYSFYVPYPFNPSQGYAAYSALTLIGAILQLPYISGGLTSFDFTY QSNGQNYEWPNQVASGTQIMAGVYNISSSPNEFTQVSLYNGFLNNGLWSYIYSFTY PYPTTHAL*
SSV NL01B.C01.01.SSV	MRKTLLALLTLSLALLMLTTPTGAITASQLGANQIVFTTNQFYIAGVSYSNEWFSAF FHGWNPIVYDVEGIYLRNISLGYVNNPNGNGYNEIFIGIGLSAYATGTLGGAGTPGQI INNAVLAGLYIVTTSSGSYYYYVGAETLQNGNPIIAKDSPPITGSLPISVQTYLYDDGN GTATAYITLYYSYGLLTYSFYVPYPFNPSQGYAAYSALTLIGAILQLPYISGGLTSFDFTY QSNGQNYEWPNQVASGTQIMAGVYNISSSPNEFTQVSLYNGFLNNGLWSYIYSFTY PYPTTHAL*
SSV M.06.0.8v2.SSV	MLLFVYLVVNNNAHPLVSYQYPYSQFAGTTMNIYLYLSNGNVVAQFSDGSFSYTYN TGISFTPYSALVMGEAPGYYQSGVNKSILFYPIPNETFSGISFNIIGSPATAVGYETIKY ATNYPIANNYISSNYAWAFAWLSSSPSFSANAYTSSSSAQISGTLQLSYTETPLGLLYI A*
SSV M.06.0.8v1.SSV	MRKYLLMGVSLFLLSLIFLNASALTASQLGANYTESSIGTKFVTAIAYSDEYQGIGPCP PYYGYWPNTIISANENIYLPNSFTYYPSVTGTDQIIVGVGIGSNGVYMPTYPAWNLN DLPNETIYAGFMFTVKPDNSYSVSYIAFGTGPNLSYINIINGNVPKGSISWLYVQEYY NQGKQTLTITWIVHYTNNSQFTYTTILSYVVYSWSALSFVATNSIGTNEFNQLPFVN GGSVSFWFSYLSNGQIYGGPGTPASGTSIYAEVYSLSKTSYANSMLSNVTSASGVTGS WSYTFSFSENGQTTYGL*

SSV M.04.0.37.SSV	MRKGLLSLTFLALLLIPIASILPSSQTYPNTWVIAFYPGSYPSGASPSNYTTFTVNFTVPNYLQTSNGYLGLVLSTFVEANEYIGGIYSGTTYLGLQIALTFVGSPNTYVLSAQVWANGNQIANYQTLVNLVPNSEAFMTFLYNYNNGNVATGALQEGSTVIDVYFPTSYSYNSVTYNVINTYGFTDPSGANPSFAIEGTYTGQPPFSDTGWDGMWINGAPEPINAVAGQSSATGYLGGTAPIPSYYAESGLTLFHNHNVFINYWYNPYAWYFSIGTPQDYSAHGLNYVQLSEGQSYNTINGLNYALYWISP*
SSV M.04.0.29.SSV	MRKYLLMGVSLFLLSLIFLNASALTASQLGANYTESSIGTKFVTAIAYSDEYQGIGPCPPYYGYWPNTIISANENIYLPNSFTYYPSVTGTDQIIVGVGIGSNGVYMPTYPAWNLNDLPNETIYAGFMFTVKPDNSYSVSYIAFGTGPNLSYINIINGNVPKGSISWLYVQEYYNQGKQTLTITWIVHYTNNSQFTYTTILSYVVYSWSALSFVATNSIGTNEFNQLPFVNGGSVSFWFSYLSNGQIYGGPGTPASGTSIYAEVYSLSKTSYANSMLSNVTSASGVTGSWSYTFSFSENGQTTYGL*
SSV M.04.0.13.SSV	MRKSLLALLTLSLALLSLLITPTIAGVIIPPSSPIPINQISSSTDSNLLGYVFEFANNGQVQPASYASISVPIPSSIPYYYNPNEYWYDNMYAFWVALSPYTIGNGASTTFLQAGVDLLENQSVKYVLLFVYLVVNNNAHPLVSYQYPYSQFAGTTMNIYLYLSNGNVVAQFSDGSFSYTYNTGISFTPYSALVMGEAPPMTQNLNGKNYTVQFYPIPNETFSGISFNIIGSPATAVGYETIKYATNYPIANNYISSNYAWAFAWLSSSPSFSANAYTSSSSAQISGTLQLSYTETPLGLLYIA*
SSV M.03.2.5.SSV	MRKYLLMGVSLFLLSLIFLNASALTASQLGANYTESSIGTKFVTAIAYSDEYQGIGPCPPYYGYWPNTIISANENIYLPNSFTYYPSVTGTDQIIVGVGIGSNGVYMPTYPAWNLNDLPNETIYAGFMFTVKPDNSYSVSYIAFGTGPNLSYINIINGNVPKGSISWLYVQEYYNQGKQTLTITWIVHYTNNSQFTYTTILSYVVYSWSALSFVATNSIGTNEFNQLPFVNGGSVSFWFSYLSNGQIYGGPGTPASGTSIYAEVYSLSKTSYANSMLSNVTSASGVTGSWSYTFSFSENGQTTYGL*
SSV M.03.0.42.SSV	MLLFVYLVVNNNAHPLVSYQYPYSQFAGTTMNIYLYLSNGNVVAQFSDGSFSYTYNTGISFTPYSALVMGEAPGYYQSGVNKSILFYPIPNETFSGISFNIIGSPATAVGYETIKYATNYPIANNYISSNYAWAFAWLSSSPSFSANAYTSSSSAQISGTLQLSYTETPLILVYIV*
SSV M.03.0.27.SSV	MRKYLLMGVSLFLLSLIFLNASALTASQLGANYTESSIGTKFVTAIAYSDEYQGIGPCPPYYGYWPNTIISANENIYLPNSFTYYPSVTGTDQIIVGVGIGSNGVYMPTYPAWNLNDLPNETIYAGFMFTVKPDNSYSVSYIAFGTGPNLSYINIINGNVPKGSISWLYVQEYYNQGKQTLTITWIVHYTNNSQFTYTTILSYVVYSWSALSFVATNSIGTNEFNQLPFVNGGSVSFWFSYLSNGQIYGGPGTPASGTSIYAEVYSLSKTSYANSMLSNVTSASGVTGSWSYTFSFSENGQTTYGL*
SSV M.16.12.SSV	MRKGLLSLTFLALLLIPIASILPSSQTYPNTWVIAFYPGSYPSGASPSNYTTFTVNFTVPNYLQTSNGYLGLVLSTFVEANEYIGGIYSGTTYLGLQIALTFVGSPNTYVLSAQVWANGNQIANYQTLVNLVPNSEAFMTFLYNYNNGNVATGALQEGSTVIDVYFPTSYSYNSVTYNVINTYGFTDPSGANPSFAIEGTYTGQPPFSDTGWDGMWINGAPEPINAVAGQSSATGYLGGTAPIPSYYAESGLTLFHNHNVFINYWYNPYAWYFSIGTPQDYSAHGLNYVQLSEGQSYNTINGLNYALYWISP*
SSV M.12.04.SSV	MRKYLLMGVSLFLLSLIFLNASALTASQLGANYTESSIGTKFVTAIAYSDEYQGIGPCPPYYGYWPNTIISANENIYLPNSFTYYPSVTGTDQIIVGVGIGSNGVYMPTYPAWNLNDLPNETIYAGFMFTVKPDNSYSVSYIAFGTGPNLSYINIINGNVPKGSISWLYVQEYYNQGKQTLTITWIVHYTNNSQFTYTTILSYVVYSWSALSFVATNSIGTNEFNQLPFVNGGSVSFWFSYLSNGQIYGGPGTPASGTSIYAEVYSLSKTSYANSMLSNVTSASGVTGSWSYTFSFSENGQTTYGL*
SSV17	MRKGLLSLTFLALLLIPIASILPSSQTYPNTWVIAFYPGSYPSGASPSNYTTFTVNFTVPNYLQTSNGYLGLVLSTFVEANEYIGGIYSGTTYLGLQIALTFVGSPNTYVLSAQVWANGNQIANYQTLVNLVPNSEAFMTFLYNYNNGNVATGALQEGSTVIDVYFPTSYSYNSVTYNVINTYGFTDPSGANPSFAIEGTYTGQPPFSDTGWDGMWINGAPEPINAVAGQSSATDNLGGTAPIPSYYAESGLTLFHNHNVFINYWYNPYAWYFSIGTPQDYSAHGLNYVQLSEGQSYNTINGLNYALYWISP*
SSV13	MRKYLLMGVSLFLLSLIFLNASALTASQLGANYTESSIGTKFVTAIAYSDEYQGIGPCPPYYGYWPNTIISANENIYLPNSFTYYPSVTGTDQIIVGVGIGSNGVYMPTYPAWNLN

	DLPNETIYAGFMFTVKPDNSYSVSYIAFGTGPNLSYINIINGNVPKGSISWLYVQEYYNQGKQTLTITWIVHYTNNSQFTYTTILSYVVYSWSALSFVATNSIGTNEFNQLPFVNGGSVSFWFSYLSNGQIYGGPGTPASGTSIYAEVYSLSKTSYANSMLSNVTSASGVTGSWSYTFSFSENGQTTYGL*
SSV Lassen	MRKSLLALLTLSLALLMLATPTGAISASQLGASQIVFTTNQFNFAGVSYSNEWISTFFHGWNPIVYDVEGIYLGNISLGYVNNPTGNGYNEIFIGIGLSAYATGTLGGAGTPGQIINNSVLAGLYIVTTSSGYYYYVGAETLQNGNLIVAKDSSPITGSLPISLQIQLYDDGNGTATAYITLYYSYGLLTYSFYVPYPFNPSNGYASYSALTLIGAVLQLPYISGGLTSFDFVYQSNGQNYEWPNQIASGTQIMAGVYSISGSVNELTQVSLYNGFLNNGLWSYIYNFSNPYPTTYAL*
SSV19	MKWPLLLFTVLLIIGFTLIARAGTISLLSTPPVNPPAYSYFYIEFQFLPTNNTPQPYAIFVGPNPNNLTEVAEGYTLSNGTGYARVPVINAQTEYVDIVWVNQNYTMFEIFPQIQNATTTVTLSANNNQGFSFSLPTWVSWVIGAVLMLIFMGVGWKFMGPAGLAIFGIFGLFIAMFFGLLPSYLMYVILFIVAIVGARILTKQLGGGEE*
SSV7	MRKSLLTLLTLSLTLLMLATPTGAISASQLGANQIVFTTNQFNIAGVSYSNEWFSLFFHGWNPIASDVENIYLRNISLGYVNNPTGNGYNEIFIGIGLSAYATGTLGGAGTPGQILNNSVLAGLYIVTTSSGYYYYVGTETLQNGNLIVAKYSPPITGSIPISVQIYLYDDGNGTATAYITLYYSSGLVTYTFYVPYPFNPSNGYASYSALTLIGALSQLPYISGGLTSFDFIYQSNGQNYEWPNQIASGTQIMAGVYNISSSVNEMTQVSLYNGFLNNGLWSYIYSFTYPFPTTHAL*
SSV6	MKWPLLLVLVIPLLAVSSLASTQNSIQIISTPPVNPPAYSYFYLEFQFSPTNNTPQPYAIFVGPSVNNLTEVAEGYTYPNGTGYARVPVINSQIEYIDVVWVNVNYTLIQIFPQTVNTTNTTTTVNIENNQGFTFSLPSWVSWVLGSVIVLIFMGIGWKFMGPAGLAVFGVVSVFLASFFGLIPSYIIYIFVFIVAVIGARVITRQFGGGEEE*
SSV5	MRKELLSLTFLALLLIPTLGILPSAQTSPNTWVIAFYPGSYPSGTSPSNFTSFTVNFTIPPFLQTSNGYLGFVLTATVPATEYIGGLYSGTVDIALQIGLIFVTQTNTYVLTAQVWTPYGTQIYGYAETIYLPANTEAVISLIYNYANGHVAIGLLQGGVTVYNVYFPTLYSHNNCVYNVTNNGGFSEPSGQNPSFAIEGTYTGSPPFNDTGWDGMWFDGAPISDINAMTGYSSATGVGGTASIPPYYAESGLTLFHNVLFNYWYNPYAWYFSIGTPQDYSAHGLHYIQLSEGKEYNTINGLNYALQWVPP*
SSV4	MRKELLSLTFLALLLIPTLGILPSAQTSPNTWVIAFYPGSYPSGTSPSNFTSFTVNFTIPPFLQTSNGYLGFVLTATVPATEYIGGLYSGTVDIALQIGLIFVTQTNTYVLTAQVWTPYGTQIYGYAETIYLPANTEAVISLIYNYANGHVAIGLLQGGVTVYNVYFPTLYSHNNCVYNVTNNGGFSEPSGQNPSFAIEGTYTGSPPFNDTGWDGMWFDGAPISDINAMTGYSSATGVGGTASIPPYYAESGLTLFHNVLFNYWYNPYAWYFSIGTPQDYSAHGLHYIQLSEGKEYNTINGLNYALQWVPP*
SSV Ragged Hills	MRKTLLALLTLSLALLMLTTPTGAITASQIGASQIVFTTNQFYIAGVSYSNEWFSAFFHGWNPIVYDVEGIYLRNISLGYVNNPNGNGYNEIFIGIGLSAYATGTLGGAGTPGQIINNAVLAGLYIVTTSSGSYYYYVGAETLQNGNPIIAKDSPPITGSLPISLQIQLYDDGNGTATAYITLYYSYGLLTYSFYVPYPFNPSQGYASYSALTLIGAILQLPYISGGLTSSILPIKVTGKTMNGRIR*
SSV3 REY 15/4	MRKELLSLTFLALLLIPTLGILPSAQTSPNTWVIAFYPGSYPSGTSPSNFTSFTVNFTIPPFLQTSNGYLGFVLTATVPATEYIGGLYSGTVDIALQIGLIFVTQTNTYVLTAQVWTPYGTQIYGYAETIYLPANTEAVISLIYNYANGHVAIGLLQGGVTVYNVYFPTLYSHNNCVYNVTNNGGFSEPSGQNPSFAIEGTYTGSPPFNDTGWDGMWFDGAPISDINAMTGYSSATGVGGTASIPPYYAESGLTLFHNVLFNYWYNPYAWYFSIGTPQDYSAHGLHYIQLSEGKEYNTINGLNYALQWVPP*
SSVK1	MRKGLLSLTFLALLLIPIASILPSSQTYPNTWVIAFYPGSYPSGASPSNYTTFTVNFTVPNYLQTSNGYLLFVLTASVEANEYSNGIYLGTTYLFLQITLDFVNSANTYEITAQVWTYTGTLISYTETPITLYPNGEAFMTLLYNYNNGNVATGALQEGSTVIDVYFPTSYSYNSVTYNVINTYGFTDPSGANPSFAIEGTYTGQPPFSDTGWDGMWINGAPEPINAVAGHSSTTDVGGTAPIPSYYAESGLTLFHNVFINYWYNPYAWYFSIGTPQDYSAHGLNYVQLSEGQSYNTINGLNYALYWISP*

SSV1	MRKSLLALLTLSLALLSFLITPSMALNSGGSPIPIYYNYYNYYSLNAEGFGFSFNNSN<br>NWVETNFISITINLPSSLPNNYQINNAYSIVVGLSPYPVSNINIFNSPLEAYVELFSNP<br>PNTYPNEIGFVVSYGSTVFYSYTTLYSSFAGTQLTITISYTGNGFGVQFSDSNGFSHSV<br>SVSSVNFVPYGALILGSLIPNGNYYYYPVGNMLPNASVNFSYTISSFTIEGNPATSVDI<br>TTLGLEGNTAIYTSSSNWFKWVSGSVVITNAVAYTYTDLARIGGSAQINYTASQLY*

Supplemental Table S3. Signal peptide predictions for fusellovirus toxin homologs.

Signal peptides were predicted using SignalP 6.0. Predicted cleavage sites and secretion probabilities are shown.

SSV	Signal Peptide	Pos.
SSV1 | NC_001338 (orf a291)	**0.99**	25/26
SSV M8v2 | MK054219 (orf 176)	**0.99**	25/26
SSV10 | MK054236 (orf 299)	**0.99**	25/26
SSVRH | NC_005360 (orf c247)	**0.99**	25/26
SSV M8v1 | MK054218 (orf 306)	**0.99**	25/26
SSV6 | NC_013587 (orf c213)	**0.99**	20/21
SSV19 | MN496305 (orf 210)	**0.99**	22/23

SSV	Signal Peptide	Pos.
SSV5 | NC_011217 (orf 32)	**0.99**	20/21
SSV7 | NC_013588 (orf a298)	**0.99**	24/25
SSV9 | NC_005361 (orf b310)	**0.99**	23/24
SSV11 | MK054237 (orf 303)	**0.99**	24/25
SSVL | KY563228 (orf b298)	**0.99**	24/25
SSV M37 | MK054217 (orf 312)	**0.99**	23/24

Supplemental Figure S1. Representative growth inhibition phenotypes of SSV1 mutants.

Halo assays were performed by spotting 3 μl of cell-free supernatants from cells transformed with SSV1 mutant constructs onto lawns of *S.solfataricus* strain S441 and incubated for 4-5 days at 80°C. 1% SDS is used to lyse *S.solfataricus*. A "+" is a spot from a culture transformed with SWT. A "-" is a spot from an uninfected culture.

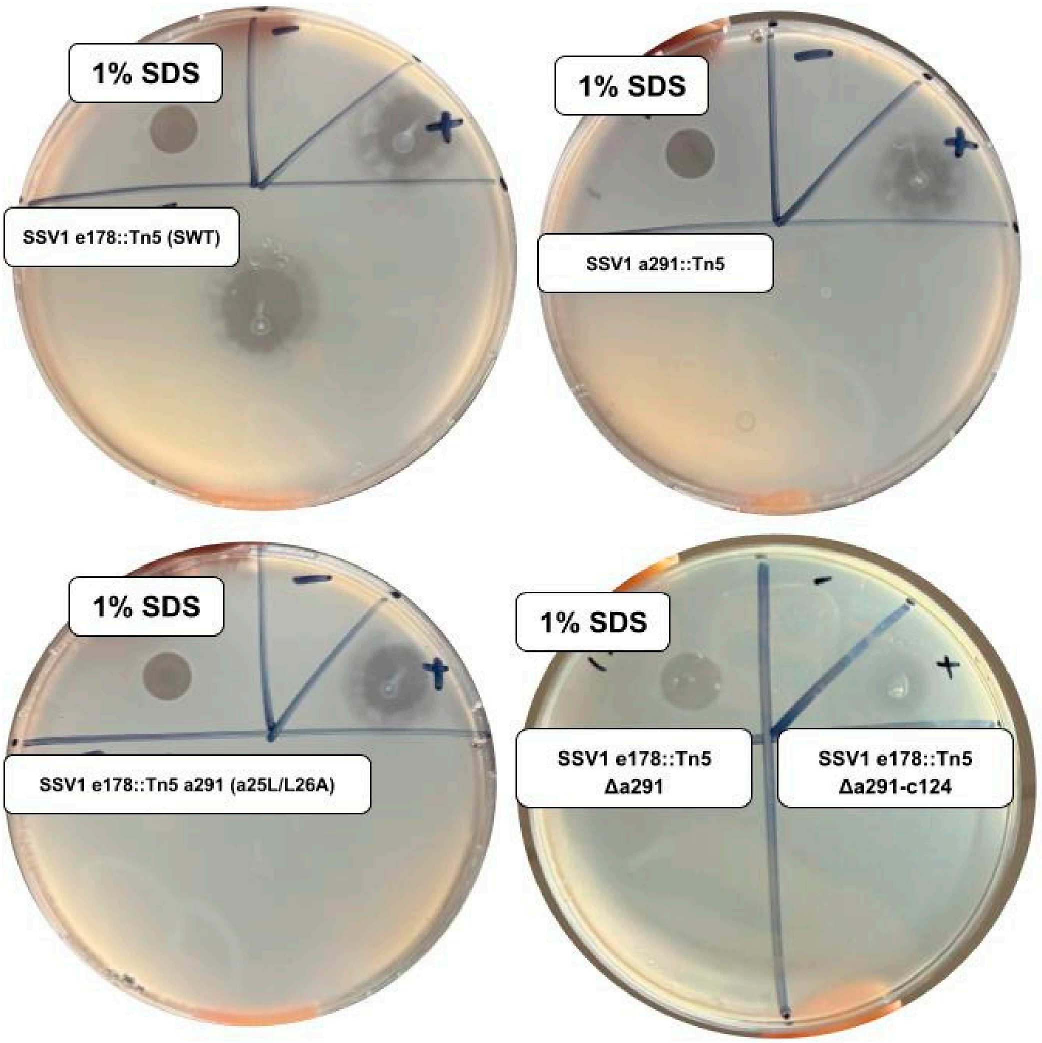

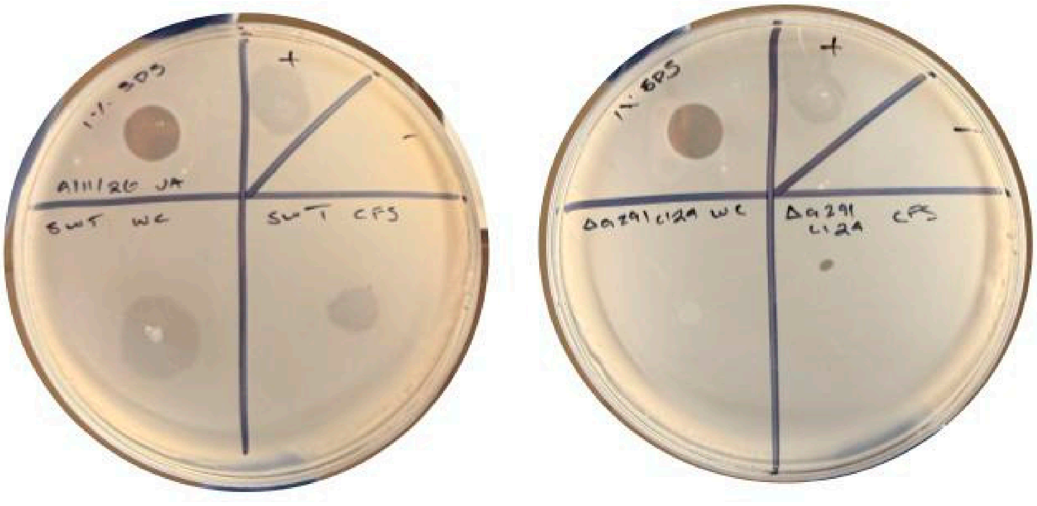
+
-
SWT WC
SWT CFS
+
-
CFS

Supplemental Figure S2. Plaque formation by SSV1 in S441.

Plaque assays were performed by spreading serial dilutions of SSV1-containing supernatants (SWT) onto semi-solid lawns of S441. Zones of clearing represent areas of infection and growth inhibition.

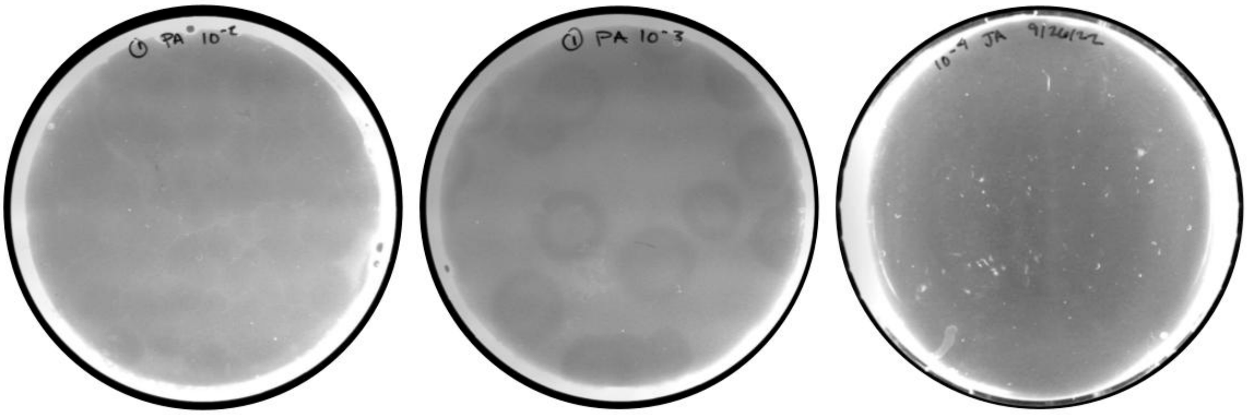

10^{-2} dilution 10^{-3} dilution 10^{-4} dilution
